# Elemental preference and atomic scale site recognition in a Co-Al-W-base superalloy

**DOI:** 10.1038/s41598-017-17456-1

**Published:** 2017-12-08

**Authors:** Yanhui Chen, Fei Xue, Shengcheng Mao, Haibo Long, Bin Zhang, Qingsong Deng, Bin Chen, Yinong Liu, Pierce Maguire, Hongzhou Zhang, Xiaodong Han, Qiang Feng

**Affiliations:** 1 Institute of Microstructure and Property of Advanced Materials, Beijing University of Technology, Beijing, 100124 China; 2 State Key Laboratory for Advanced Metals and Materials, University of Science and Technology Beijing, Beijing, 100083 China; 3 School of Mechanical and Chemical Engineering, The University of Western Australia, Perth, WA 6009 Australia; 4 School of Physics and Centre for Research on Adaptive Nanostructures and Nanodevices (CRANN), Trinity College Dublin, Dublin 2, Republic of Ireland

## Abstract

Using state-of-the-art atomic scale super energy dispersive X-ray spectroscopy and high angle annular dark field imaging this study reveals the elemental partitioning preference between the γ′ and γ phases in a Co-Al-W-Ti-Ta superalloy and the site preference of its alloying elements in the ordered L1_2_ γ′ phase. A semi-quantitative analysis of atomic column compositions in the ordered L1_2_ γ′ structure is provided. Co atoms were found to occupy the {1/2, 1/2, 0} face-center positions whereas Al, W, Ti and Ta atoms prefer to occupy the {0, 0, 0} cube corner positions in the L1_2_ γ phase. These findings agree well with predictions from first principles simulations in the literature.

## Introduction

Co-Al-W-based superalloys derive their high temperature strength largely from a γ′-Co_3_(Al, W) precipitate phase^1–3^. The γ′ phase has an ordered L1_2_ structure which is more resistant to dislocation movement than its parent disordered FCC phase, thus exhibiting a higher strength. The L1_2_ structure was first identified in Ni_3_Al^4^ alloy in 1937 and later discovered in other alloys such as Cu_3_Au^5^ alloy. The Ni-based and Co-based superalloys are typical L1_2_ containing alloys and they derive their high temperature strengths largely from their L1_2_ precipitates. The Ni-based superalloys have been used in turbine engines^6^. Ti and Ta are often added in these alloys to further improve the structural stability of γ/γ′ two-phase microstructures by increasing the γ′ volume fraction^7–11^. It has been shown that the γ′ strengthened Co-Al-W-Ti-Ta superalloys display improved structural stability, exceeding both conventional Co-based superalloys and the first-generation Ni-base single-crystal superalloys in creep life at around 1000 °C^11,12^.

The superior creep resistance of Co-Al-W-Ti-Ta superalloys derives from the intrinsic high resistance to dislocation shear of the L1_2_ γ′ precipitate. Due to the ordered structure, the Burger’s vector of a full dislocation in the L1_2_ γ′ phase is twice the magnitude of that in the FCC γ phase, i.e., ${b}_{\gamma ^{\prime} }=2{b}_{\gamma }=2[\frac{a}{2} < 110 > ]$. This implies that a *b*_*γ*_ = a/2 <110> full dislocation in the FCC γ phase functions only as a partial dislocation in the γ′ phase and that dislocations in the γ′ phase always move in pairs of different *m* <110> and *n* <112> combinations. The passing of these partial dislocation pairs produces different forms of stacking faults (SFs) and anti-phase boundaries (APBs)^11,12^. Therefore, stacking fault energy and anti-phase boundary energy directly influence the activation and slipping of these dislocations and thus the mechanical strength of the γ′ precipitates^13–15^.

Stacking fault energy and anti-phase boundary energy are in turn determined by the elemental structure of the atomic plane in question, particularly for A_3_B type alloys containing other alloying elements, for example the addition of Ti and Ta in the Co-Al-W alloy. Therefore, knowledge of lattice site occupancy of alloying elements in γ′-Co-Al-W-Ti-Ta is important for developing high strength and creep resistant Co-based superalloys.

Much effort has been made in theoretical analysis, mostly by means of first principles simulation, to predict lattice site preference of alloying elements in the γ′ phase^16–18^. It is generally predicted that Ti has equal preference for the face-centered {1/2, 1/2, 0} and cube-corner {0, 0, 0} sites in the L1_2_ structure^17^ and that Ta has a preference for the {0, 0, 0} sites^18^.

However, to date there has not been direct experimental evidence to support or verify such predictions. One possible experimental technique to use is atomically resolved chemical analysis using transmission electron microscopy (TEM)^19,20^. Resolving atomic site preferences of elements in a thin region of the Co-Al-W-Ti-Ta alloy is challenging because of the weak signals and spatial resolution of conventional TEM equipment and energy-dispersive X-ray spectroscopy (EDS) detectors. These limitations can be overcome by using a spherical aberration (Cs) corrected probe and super energy-dispersive X-ray spectroscopy (super-EDS) detector^21,22^. In the present work, we investigated the elemental partitioning between the atomic columns in the γ′ and γ phases in a Co-Al-W-Ti-Ta alloy and the site preferences of Co, Al, W, Ti and Ta in the L1_2_ γ′ structure using high resolution high angle annular dark field (HR-HAADF) and atomic super-EDS techniques.

## Results and Discussions

**Figure 1:**
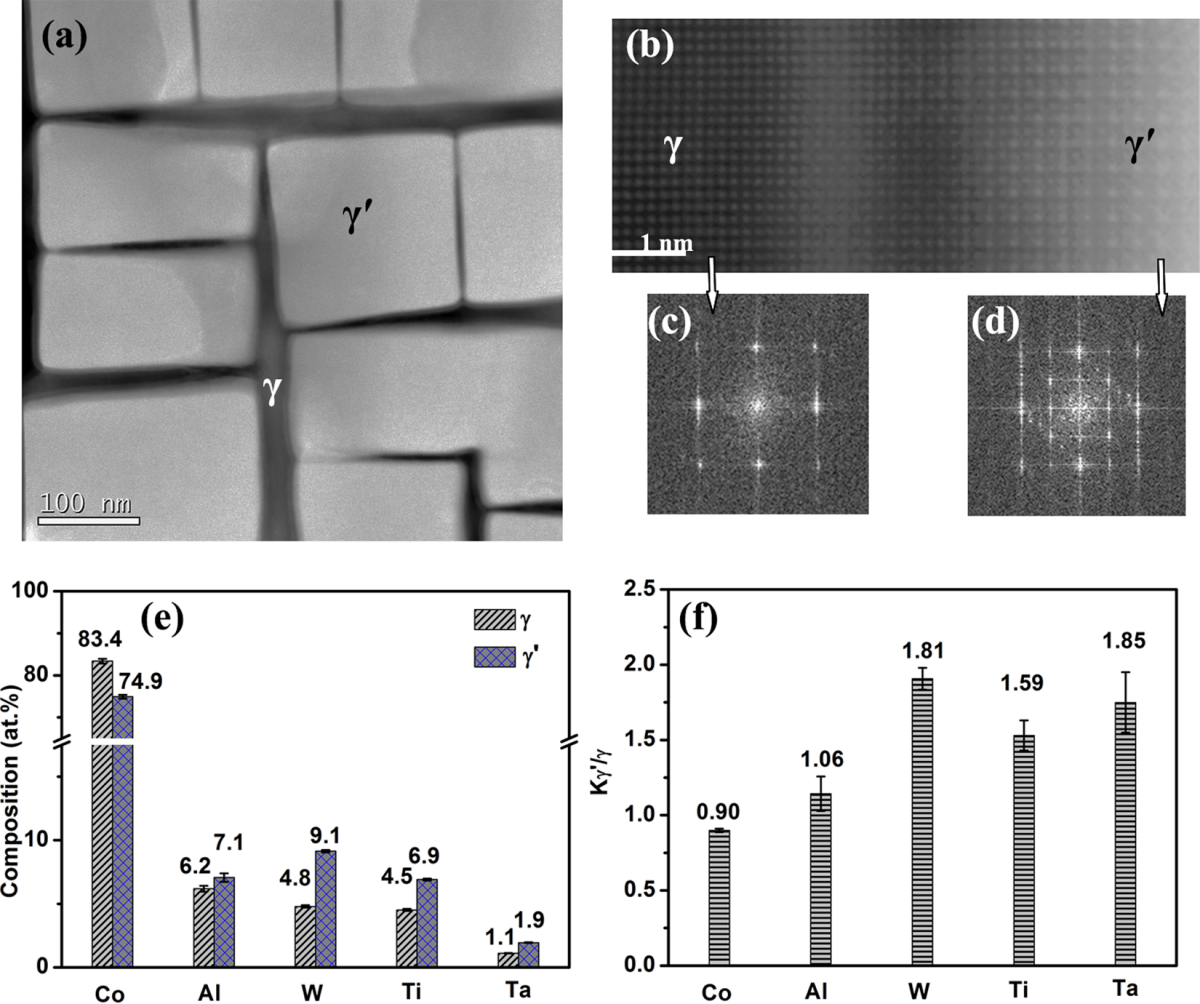
Composition analysis of the γ′ and γ phases. (**a**) HAADF image of the microstructure of the Co-7Al-8W-4Ti-1Ta single crystal alloy after ageing treatment. (**b**) High resolution HAADF image of a region across a γ/γ′ boundary. (**c**) FFT pattern of the γ phase region. (**d**) FFT pattern of the γ′ region. (**e**) Concentrations of Co, Al, W, Ta and Ti in the γ and γ′ phases. (**f**) Element partitioning coefficients K_γ′/γ_ of the alloying elements.

Figure 1 shows the microstructure of the Co-7Al-8W-4Ti-1Ta alloy single crystal after the ageing treatment. The γ′ phase is in cuboidal shapes which are all aligned along <001> directions, whereas the γ phase forms a continuous network of orthogonally connected narrow channels in between these γ′ cuboids (Fig. 1(a)). Figure 1(b) shows a high-resolution HAADF image of a region containing both the γ and γ′ phases, and Fig. 1(c) and (d) show the Fast Fourier Transform (FFT) patterns of the two regions, respectively. It is clear that the γ′ phase is well ordered and the γ phase is not.

Quantitative EDS analysis of the composition of the two phases was conducted. Figure 1(e) shows the concentrations of the five elements in the γ and γ′ phases. Figure 1(f) shows the partitioning coefficients, K_γ′/γ,_ (defined as the ratio of the concentration in γ′ to that in γ) of the elements between the two phases. It is seen that Co has a lower concentration in the γ′ phase, apparently to meet the stoichiometry requirement for the L1_2_ structure (75 at.%). This gives a K_γ′/γ_ value of 0.9 for Co. Al shows little preference between the two phases with a small partitioning coefficient of 1.06. By contrast, W, Ti and Ta show clear preference for the γ′ phase with partitioning coefficients of 1.81, 1.59 and 1.85, respectively. Given that the Co content in the γ′ phase is ~75 at.% of that expected in its theoretical form of Co_3_(Al, W), i.e. the stoichiometry of the L1_2_ structure, the {1/2, 1/2, 0} site is expected to contain 100 at.% Co atoms and the {0, 0, 0} site contains all the other elements of Al, W, Ti and Ta. The partitioning of W, Ti and Ta in the {0, 0, 0} sites of the L1_2_ structure implies that the bonding energies of Co-W, Co-Ta and Co-Ti bonds are higher than that of Co-Co bond, as predicted by Saal and Wolverton in their density functional theory (DFT) calculations^23^.

**Figure 2:**
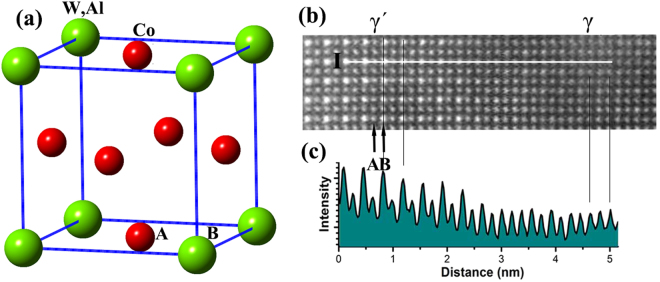
Structure of the γ′-Co_3_(Al, W) phase in the Co-7Al-8W-4Ti-1Ta alloy. (**a**) Atomic model of L1_2_ structure. (**b**) HAADF image of a region containing both γ′ and γ phases. (**c**) HAADF image intensity profile along line I.

Figure 2 shows HAADF analysis of a region containing both the γ and γ′ phases in the Co-7Al-8W-4Ti-1Ta alloy in the [001] zone axis. Figure 2(a) shows the atomic structure of an L1_2_ unit cell. The {1/2, 1/2, 0} positions are labelled as the A sites (the red balls) and {0, 0, 0} positions are denoted the B sites (the green balls). Figure 2(b) shows a high-resolution HAADF image in the [001] zone axis of a region containing both γ and γ′ phases. In a HAADF image, the brightness of a spot is roughly proportional to Z^1.6–1.7^, where Z is the atomic number^24^, or in this case the average atomic number of the atomic column in the direction of the electron beam. In this image, the bright spots are the {0, 0, 0} sites (site B) and the dark spots which are three times more populous than the bright spots are the {1/2, 1/2, 0} sites (site A). The intensity ratio of two adjacent atoms in a HAADF image gives a qualitative comparative measure of the aggregated atomic weights of the columns without distinction between specific elements. By comparison, the EDS mapping provided by the Super-EDS technique gives a semi-quantitative description of the actual elements and their partitions between the columns.

Figure 2(c) shows the intensity ratio profile of the atomic columns (in the direction perpendicular to the page plane) along line “I” in the HAADF image of the interface region between the γ and γ′ phases shown in Fig. 2(b). The intensity ratio of each column is determined as relative to that of its left adjacent column. It is seen that in the γ′ phase region the spot intensity is much more distinctive between the A and B sites and that the B site has more heavy elements than the A site, reflecting the ordered structure of the γ′ phase. By contrast, in the solid solution γ phase region the spot intensity is much more uniform, indicating no site preference for the elements.

**Figure 3:**
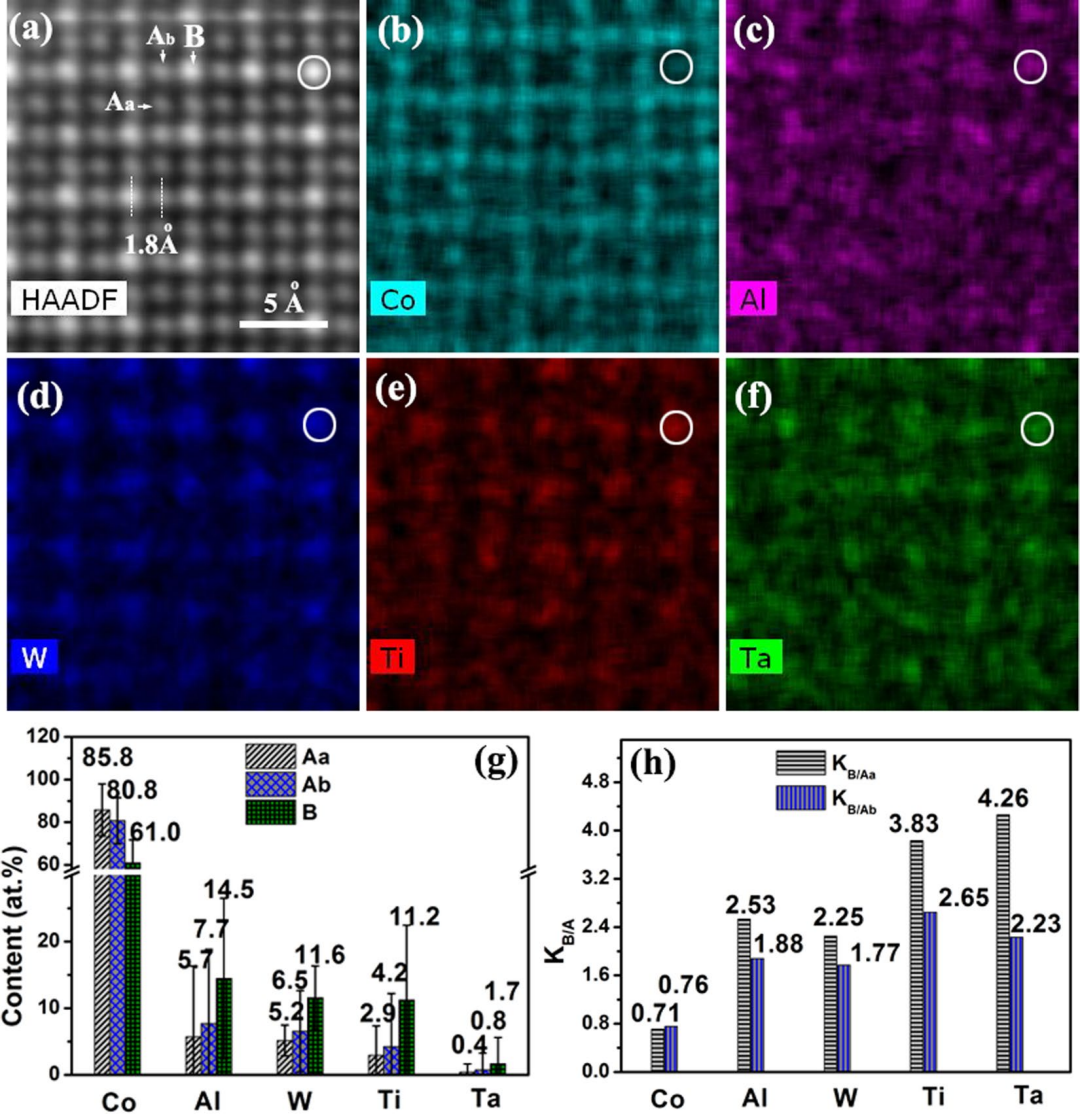
Atomic scale elemental mapping and quantification of the γ′ phase in the Co-Al-W-Ti-Ta alloy. (**a–e**) High resolution HAADF imaging and elemental mapping. (**g**) Elemental contents of atomic columns at sites A_a_, A_b_ and site B. (**h**) Element partitioning coefficients K_B/Aa_ and K_B/Ab_ for different elements.

While the HAADF image technique can detect the difference in average atomic number between different atomic columns, it is unable to detect which individual element may have contributed to the average atomic number in an atomic column. For this, atomically resolved EDS mapping analysis was carried out for the alloying elements in the γ′ phase in the Co-7Al-8W-4Ta-Ti alloy, as presented in Fig. 3. Figure 3(a) is a HAADF image of the γ′ phase, as viewed from the [001] direction. The dark sites are the A sites and the bright sites are the B sites. Based on the surrounding environment when viewed from the [001] direction, the A sites can be further divided into two types, the A_a_ sites, which are surrounded by four other A sites and the A_b_ sites, which are surrounded by two A sites and two B sites. It needs to be noted that the A_a_ and A_b_ sites are totally equivalent in the 3-dimentional environment in the lattice. They are different here only with respect to the viewing direction, in which the composition analysis was conducted. Figure 3(b)–(f) show the partitioning maps of Co, Al, W, Ti and Ta. One B site is encircled as a landmark for all element map images. It is apparent that Co has a strong presence at the A sites (Fig. 3(b)). These sites have a low intensity in the HAADF image shown in Fig. 3(a). By comparison, Al, W, Ti and Ta have tendencies to occupy the B sites.

Figure 3(g) shows elemental compositions of the [001] atomic columns at the A sites (including A_a_, A_b_) and the B sites. For the analysis, six site A_a_ atomic columns, twelve site A_b_ atomic columns and six site B atomic columns were analyzed, and the data shown in Fig. 3(g) are the averages of these measurements. Figure 3(h) shows site preference coefficient of site B to site A for the five elements. The site preference coefficients indicate that Al, W, Ti and Ta all have preference to occupy the B sites whereas Co prefers to occupy the A sites. Values of K_B/Aa_ on Al, W, Ti and Ta are higher than values of K_B/Ab_, i.e., the A_b_ site exhibits less partitioning than the A_a_ site relative to the B site. This is attributed to the higher level of signal contamination of the Ab site due to its proximity to the B site.

Co is found to have a preference for the A site. Its concentration is measured to be 85.8 at.% at the A_a_ site and 80.8 at.% at the A_b_ site. The lower concentration at the A_b_ site is apparently due to the signal contamination from the B site and thus is considered a less accurate representation of the A site (the A_a_ and A_b_ sites are identical sites in the crystal structure and thus have the identical composition). Therefore, the A_a_ site composition is considered a more accurate measure of the composition of the A site, though still not a perfect representation due to the inevitable signal contamination of the technique used. However, a significant amount of Co is also detected at the B site, with a detected concentration of 61.0 at.%. In an ideal Co_3_X L1_2_ structure the A_[001]_ column is expected to be 100% Co and the B_[001]_ column is expected to have no Co. The Co content being lower than purity at the A site is due to signal contamination from the surrounding columns caused by the spread of the electron beam as it penetrates the sample^25^. Similarly, the detection of Co at the B site is also due to column signal contamination. Considering that a B_<001>_ column is always surrounded by four A_<001>_ columns, the level of contamination is expected to be higher than that of the A_<001>_ columns, thus the high Co content measured.

To verify this, column compositions of site A_a_ and site A_b_ were measured separately, as presented in Fig. 3(g). Despite the total equivalence in the L1_2_ structure, the two sites show clear differences in element concentrations, with lower Co concentrations and higher concentrations of other elements at the A_b_ site than A_a_ site. This is apparently related to the closer neighboring relationship of A_b_ to the B sites.

A number of factors including beam spread, probe channeling, image delocalization, thermal scattering^25,26^, sample alignment and thickness^26,27^ limit the spatial resolution of composition quantification. In our experiment, the beam convergence angle is about 30 mrad and the beam spot size is about 2 Å. As seen in Fig. 3(a), the average A/A and A/B site distance is 1.8 Å which is smaller than the beam size. The sample thickness is ~70 nm. Therefore, while the spatial resolution is sufficient at the top surface of the sample foil for composition determination of a single column, it is inevitable that the composition quantification is always contaminated by signals from the neighboring atomic columns when the penetration depth increases. It is evident in Fig. 3(g) that the composition of the A_b_ columns is closer to that of the B site columns due to the influence of beam-spread induced signals from the adjacent B columns. In this regard, the measured composition of the A_a_ site is considered less contaminated and thus a more reliable measurement.

Al, W, Ti and Ta all show preferences for the B site. Saal *et al*. conducted DFT calculations and suggested that Co_3_Al and Co_3_W are less stable L1_2_ phases^23^. In this regard, the actual γ′ phase in the Co-7Al-8W-4Ti-1Ta alloy can be described as Co_3_(Al, W, Ti, Ta), i.e., an ordered L1_2_ structure between the A and B sites with a disordered solid solution of W, Al, Ti and Ta at the B site. Neglecting the Co content measured, the B site composition may be estimated to be 14.5 at.%W, 11.6 at.%Al, 14.2 at.%Ti and 1.7 at.%Ta. This gives a weighted average atomic number of 32.26 for the B site. For the A site, the measurement from the A_a_ site is used, because of its relatively lower signal contamination, and the composition is estimated to be 5.7 at.%W, 5.2 at.%Al, 2.9 at.%Ti and 0.4 at.%Ta. This gives a weighted average atomic number of 28.99 for the A site. In this regard, the average atomic number ratio between the B site and the A site is estimated to be 32.26/28.99 = 1.11, according to the Super-EDX measurements.

On the other hand, the average atomic number ratio may also be estimated from HAADF measurement. The HAADF intensity ratio of the B site to the A_a_ site is 2.2, as determined in the centre of a γ′ phase region in a HAADF image. It has been suggested that the HAADF intensity (I) of an atomic column and its weighted average atomic number (Z) follow the relationship of I ∝ Z^1.6–1.7^. Using this relationship, the average atomic number ratio between the B site and the A_a_ site is estimated to be 1.59–1.64. The ratio is closer to a nominal Co_74.9_Al_7.1_W_9.1_Ti_6.9_Ta_1.9_ obtained from γ′ phase and it has an ideal atomic number ratio of 1.56. It is seen that the two values determined by the two methods are different, but both are larger than 1, indicating the segregation of heavy elements to the B sites. It can be conclude that the average atomic numbers obtained from the EDS atomic scale mapping method are less accurate than those obtained from a HAADF image.

Atom Probe Tomography (APT) is another material analysis technique offering extensive capabilities for 3D imaging and chemical composition measurements at the atomic scale. This technique has spatial resolutions of typically 1–3 Å in depth and 3–5 Å in lateral directions. The super-EDS technique differs from APT in several aspects. First, the super-EDS in TEM technique used in this study has a higher lateral spatial resolution of ~0.7 Å when used in a Cs-corrected TEM. This is more suited for composition analysis of atomic columns such as those which are separated by 1.8 Å in our alloy. Second, the 3D tomography needs a reconstruction process that introduces secondary errors, whereas the Super-EDS in TEM technique quickly generates direct elemental mapping in real time. Third, the APT technique is more advantageous than the Super-EDS method by not suffering from signal contamination caused by beam spread. The APT method conducts composition analysis layer by layer by progressively ablating the sample surface with laser or high voltage pulsed ion beam, thus avoiding the problem of beam spreading and minimizing signal contamination from adjacent atomic columns^28,29^.

## Conclusions

Using atomic scale super energy dispersive X-ray spectroscopy and high angle annular dark field imaging we studied elemental partitioning in the γ′/γ structure and atomic site preference in a Co-Al-W-Ti-Ta superalloy. The Super-EDS technique provides a semi-quantitative analysis of elemental partitioning. Co was found to have a site preference for the {1/2, 1/2, 0} sites whereas Al, W, Ti and Ta have preferences for the {0, 0, 0} positions of the L1_2_ unit cell. These findings are consistent with the findings of first principles investigations reported in the literature and this study provided direct experimental evidence for the first time.

## Methods

The experimental alloy has a nominal composition of Co-7Al-8W-4Ti-1Ta (at.%)^11^. The alloy was fabricated into single crystal bars of 15 mm in diameter and 150 mm in length by means of directional solidification using the conventional Bridgman method. The single-crystal ingot was solution treated at 1270 °C for 24 h in a flowing Ar atmosphere, and then aged at 900 °C for 50 h to encourage γ′ phase formation followed by air cooling. TEM samples were prepared by means of a twin-jet electrochemical polishing method. The alloy we used is a highly crystalline single crystal with the cubic shape γ′ phase about 200 nm and we chose the center in a 200 nm γ′ phase to get EDS mapping results. The thickness measured in our experiments is about 50–70 nm by the two beam CBED method. The region we measured in the γ′ phase did not contain γ′ phase lying beneath the ordered phase. TEM, HAADF and EDS mapping experiments were carried out using an FEI Titan G2 60–300 microscope operated at 300 kV. Quantification was performed from average data of 6 A_a_, 12 A_b_ and 6 B columns in one image. For quantitative EDS analysis, the power law method was applied to remove the background. The K series peaks were chosen for Al, Ti and Co and the L series peaks were chosen for W and Ta. Phase partitioning coefficient K_γ′/γ_ and site preference partitioning coefficient K_B/A_ were defined as the concentration ratios of an element in γ′ to γ phase at sites A and B, respectively.
